# Gene Profiling in Patients with Systemic Sclerosis Reveals the Presence of Oncogenic Gene Signatures

**DOI:** 10.3389/fimmu.2018.00449

**Published:** 2018-03-06

**Authors:** Marzia Dolcino, Andrea Pelosi, Piera Filomena Fiore, Giuseppe Patuzzo, Elisa Tinazzi, Claudio Lunardi, Antonio Puccetti

**Affiliations:** ^1^Department of Medicine, University of Verona, Verona, Italy; ^2^Immunology Area, Pediatric Hospital Bambino Gesù, Rome, Italy; ^3^Department of Experimental Medicine – Section of Histology, University of Genova, Genova, Italy

**Keywords:** systemic sclerosis, cancer, gene expression, miRNA, gene module, protein–protein interaction network

## Abstract

Systemic sclerosis (SSc) is a rare connective tissue disease characterized by three pathogenetic hallmarks: vasculopathy, dysregulation of the immune system, and fibrosis. A particular feature of SSc is the increased frequency of some types of malignancies, namely breast, lung, and hematological malignancies. Moreover, SSc may also be a paraneoplastic disease, again indicating a strong link between cancer and scleroderma. The reason of this association is still unknown; therefore, we aimed at investigating whether particular genetic or epigenetic factors may play a role in promoting cancer development in patients with SSc and whether some features are shared by the two conditions. We therefore performed a gene expression profiling of peripheral blood mononuclear cells (PBMCs) derived from patients with limited and diffuse SSc, showing that the various classes of genes potentially linked to the pathogenesis of SSc (such as apoptosis, endothelial cell activation, extracellular matrix remodeling, immune response, and inflammation) include genes that directly participate in the development of malignancies or that are involved in pathways known to be associated with carcinogenesis. The transcriptional analysis was then complemented by a complex network analysis of modulated genes which further confirmed the presence of signaling pathways associated with carcinogenesis. Since epigenetic mechanisms, such as microRNAs (miRNAs), are believed to play a central role in the pathogenesis of SSc, we also evaluated whether specific cancer-related miRNAs could be deregulated in the serum of SSc patients. We focused our attention on miRNAs already found upregulated in SSc such as miR-21-5p, miR-92a-3p, and on miR-155-5p, miR 126-3p and miR-16-5p known to be deregulated in malignancies associated to SSc, i.e., breast, lung, and hematological malignancies. miR-21-5p, miR-92a-3p, miR-155-5p, and miR-16-5p expression was significantly higher in SSc sera compared to healthy controls. Our findings indicate the presence of modulated genes and miRNAs that can play a predisposing role in the development of malignancies in SSc and are important for a better risk stratification of patients and for the identification of a better individualized precision medicine strategy.

## Introduction

Systemic sclerosis (SSc) is a rare, chronic disease characterized by three pathogenetic hallmarks: vasculopathy, dysregulation of the immune system, and increased extracellular matrix deposition in the skin and internal organs, leading to extended fibrosis and to a remarkable heterogeneity in clinical features and course of the disease, resulting in high morbidity and mortality (1).

Several environmental factors alongside genetic susceptibility and epigenetic mechanisms contribute to the onset of the disease (2–5).

Increased frequency of a few types of cancer, namely breast, lung, and hematologic malignancies in SSc has been reported (6), and it seems to be associated with the presence of particular autoantibodies (7). Moreover, it is worthwhile mentioning that SSc may also be a paraneoplastic disease (8, 9), indicating a strong link between cancer and scleroderma.

Given the high incidence of tumors in patients with SSc, we wanted to assess whether this phenomenon may be supported by a particular gene modulation that can favor cancer development in patients with SSc. Thus, we analyzed transcriptional profiles of peripheral blood mononuclear cells (PBMCs) obtained from patients with SSc to evaluate the presence of modulated genes that are involved in signaling pathways associated with malignancy. Moreover, through a complex network analysis, we verified which pathways could play a pivotal role in cancer development in SSc patients.

Besides environmental and genetic factors, epigenetic mechanisms, such as microRNAs (miRNAs), are believed to play a central role in the pathogenesis of the disease.

microRNAs are a class of small non-coding RNAs that bind 3’ untranslated region of messenger RNAs (mRNAs), negatively regulating target gene expression by either the repression of translation or degradation of target mRNAs (10). miRNAs are considered an important class of epigenetics regulators in many basic cellular processes as well as in the vast majority of diseases, including cancer and autoimmunity (11, 12). Little is known about the role of dysregulated expression of miRNAs in the pathogenesis of SSc (13). An altered expression of profibrotic and/or antifibrotic miRNAs has been suggested as an important factor in the development of fibrosis in SSc patients (14). Moreover, increased evidence suggests that serum miRNA levels may be promising biomarkers for the diagnosis, prognosis, and therapeutic approach in SSc (15). Serum levels of miR-21 and miR-92a have been found significantly higher in SSc samples compared to normal controls (16, 17). Their function is implicated in inflammation, in regulation of immune cells (18) and in favoring fibrosis (16, 17).

Since particular miRNAs have been associated with malignancies, we aimed at evaluating whether transcriptional profiles and dysregulation of particular miRNAs are shared by cancer and SSc.

## Materials and Methods

### Patients

We enrolled 30 patients affected by SSc, attending the Unit of Autoimmune Disease at the University Hospital of Verona, and 30 sex and age matched healthy controls. All patients fulfilled the ACR/Eular classification criteria for SSc (19). The distinction between limited (lSSc) and diffuse cutaneous SSc (dSSc) was performed according to the criteria proposed by LeRoy et al. (20). Fifteen of the 30 patients were affected by lSSc and 15 by dSSc. The clinical and demographic features of the patients enrolled in the study are summarized in Table 1.

**Table 1:** Clinical and demographic features of the patients enrolled in the study.

Demographic and clinical features		Systemic sclerosis patients	
		lSSc	Diffuse cutaneous SSc
Patients		15	15
Male/female		2/13	1/15
Mean age (years)		56 ± 15	54 ± 12

Laboratory findings	ANA	14 (93%)	15 (100)
	Anti-centromere	9 (60%)	3 (20%)
	Scl-70	1 (6%)	11 (73%)

Lung involvement	Interstitial disease	4 (26%)	8 (53%)
	Pulmonary arterial hypertension	1 (6%)	2 (13%)

Skin involvement	mRSS	8 ± 3	14 ± 8
	Digital ulcers	5 (33%)	7 (46%)

Video Capillaroscopy	Early	1 (6%)	4 (26%)
	Active	8 (53%)	5 (33%)
	Late	6 (40%)	6 (40%)

Kidney involvement		0 (0%)	1 (6%)
Gastro-intestinal involvement		9 (60%)	14 (93%)

Samples obtained from 10 patients with lSSc, 10 patients with dSSc, and 10 healthy controls were used for the gene expression analysis, whereas samples obtained from all the patients and controls were used for the microRNA study. Blood samples were collected from patients with active disease and in the absence of immunosuppressive therapies.

A written informed consent was obtained by all the participants to the study and the study protocol was approved by the Ethical Committee of the Azienda Ospedaliera Universitaria Integrata di Verona. All the investigations have been performed according to the principles contained in the Helsinki declaration.

### Gene Expression Analysis

Blood samples were collected in BD Vacutainer K2EDTA tubes using a 21-gage needle. PBMC were obtained upon stratification on Lympholyte^®^ cell separation density gradient (Cedarlane, Burlington, Canada). Total RNA extraction from PBMC was performed with miRNeasy mini kit following manufacturer’s protocol (Qiagen GmbH, Hilden, Germany). cRNA preparation, samples hybridization, and scanning were performed following the Affymetrix (Affymetrix, Santa Clara, CA, USA) provided protocols, by Cogentech Affymetrix microarray unit (Campus IFOM IEO, Milan, Italy). All samples were hybridized on Human Clariom D (Affymetrix) gene chip and were analyzed using the Transcriptome Analysis Console (TAC) 4.0 software (Applied Biosystem, Foster City, CA USA by Thermo Fisher Scientific, Waltham, MA, USA). The Signal Space Transformation (SST)-Robust Multi-Array Average algorithm (RMA) were applied to background-adjust, normalize, and log-transform signals intensity.

Relative gene expression levels of each transcript were validated applying a one-way analysis of variance (ANOVA) (*p* ≤ 0.01) and multiple testing correction. Genes that displayed an expression level at least 1.5 fold different in the test sample versus control sample (*p* ≤ 0.01) were submitted both to functional classification, using the Gene Ontology (GO) annotations, and to Pathway analysis, employing the Panther expression analysis tools^1^ (21). The enrichment of all pathways and functional classes associated to the differentially expressed genes compared to the distribution of genes included on the Clariom D microarray was analyzed and *p* values ≤0.05, calculated by the binomial statistical test, were considered as significant enrichment.

### Protein–Protein Interaction (PPI) Network Construction and Network Clustering

The Search Tool for the Retrieval of Interacting Genes (STRING version 10.5^2^) is a web-based database which comprises experimental as well as predicted interaction and covers >1,100 completely sequenced organisms (22). DEGs were mapped to the STRING database to detect protein–protein interactions (PPI) pairs that were validated by experimental studies (23) and the corresponding PPI network was constructed. A score of ≥0.7 for each PPI pair was selected to design the PPI network.

To detect high-flow areas (highly connected regions) of the network, we used the CFinder software tool, based on the Clique Percolation Method (CPM) (24). Finally, Cytoscape software (25) was used to define the topology of the built networks.

### Cell-Free microRNA Expression in SSc Sera

To isolate cell-free circulating miRNA (cf-miRNA) in serum, we used miRNeasy serum/plasma kit (Qiagen, GmbH, Hilden, Germany). For all the samples, RNA was extracted from 200 µL of serum in accordance with manufacturer’s instructions. In order to minimize the technical variation between samples in down-stream PCR analysis we added, for all isolations, 0.90 fmol of spike-in non-human synthetic miRNA cel-39-3p into the sample after the addition of the lysis/denaturant buffer to the serum. After extraction, RNA was eluted in 14 µL of nuclease-free water.

Mature miRNA expression was assayed by TaqMan^®^ Advanced miRNA assays chemistry (Applied Biosystems, Foster City, CA, USA). Briefly, 2 µL of serum RNA was reverse transcribed and pre-amplified with TaqMan^®^ Advanced miRNA cDNA synthesis kit following manufacturer’s instructions (Applied Biosystems, Foster City, CA, USA). Pre-amplified cDNA was diluted 1/10 in nuclease-free water and 5 µL of diluted cDNA for each replicate were loaded in PCR. 20 µL PCR reactions were composed by 2× Fast Advanced Master Mix and TaqMan^®^ Advanced miRNA assays for hsa-miR-126-3p (477887_mir), hsa-miR-21-5p (477975_mir), hsa-miR-92a-3p (477827_mir), hsa-miR-155a-5p (477927_mir), and hsa-miR-16-5p (477860_mir). Since established consensus house-keeping miRNAs for data normalization are lacking for serum miRNAs, we used cel-39-3p expression (478293_mir) to normalize miRNA expression. Real time PCR were carried out in triplicate on a QuantStudio 6 Flex instrument (Applied Biosystems, Foster City, CA, USA). Expression values were obtained by ΔCt method using QuantStudio Real-time PCR system software v. 1.3.

## Results

### Gene Array Analysis

In order to gain new insights into the pathogenesis of SSc, we compared the gene expression profiles of PBMC samples obtained from 10 lSSc and 10 dSSc patients with 10 PBMC samples obtained from age and sex matched healthy donors. Raw data have been deposited in the *ArrayExpress* database at EMBL-EBI^3^ under accession number E-MTAB-6531.

When we analyzed lSSc samples, we found that 829 differently expressed genes (DEGs) satisfied the Bonferroni-corrected *p* value criterion (*p* ≤ 0.01) and the fold change criterion (FC ≥ |1.5|), displaying robust and statistically significant variation between SSc and healthy controls samples. In particular, 736 and 93 transcripts resulted to be over- and underexpressed, respectively. The complete list of modulated genes can be found in Table S1 in Supplementary Material.

When the same Bonferroni-corrected *p* value and fold change criteria were used, 456 transcripts were differentially modulated in dSSc patients compared to healthy controls, 327 and 129 transcripts resulted to be up- and downregulated, respectively (Table S2 in Supplementary Material).

The Gene Ontology analysis of modulated genes in lSSc patients, showed that a large number of the modulated transcripts can be ascribed to biological processes that may play a role in SSc, including: apoptotic process, cell proliferation, growth factor and growth factor binding, inflammatory response, immune response, angiogenesis, endothelial cell activation, cell adhesion, and extracellular matrix organization process.

Moreover, a large number of modulated genes were ascribed to well-known signaling pathways encompassing Type I interferon, epidermal growth factor (EGF) receptor, transforming growth factor (TGF)-beta, interleukin, Wnt, glycolysis, platelet derived growth factor (PDGF), FAS, and Phosphoinositide 3 (PI3)-kinase signaling pathway.

Table 2 shows a selection of DEGs within the above-mentioned categories and also includes public gene accession numbers and fold changes.

**Table 2:** Selection of modulated genes in lSSc patients versus healthy controls.

ID	Gene symbol	Description	Fold change	*p*-Value	Public gene IDs
**Inflammatory response**					
TC0400011053.hg.1	CXCL10	Chemokine (C-X-C motif) ligand 10	5.20	0.0043	NM_001565
TC1100009225.hg.1	CXCR5	Chemokine (C-X-C motif) receptor 5	2.54	0.0036	NM_001716
TC1100008341.hg.1	FOLR2	Folate receptor 2	1.90	0.0065	NM_000803
TC0200008675.hg.1	IL18RAP	Interleukin 18 receptor accessory protein	-2.13	0.0027	NM_003853
TC1000009691.hg.1	IL2RA	Interleukin 2 receptor, alpha	2.35	0.0010	NM_000417
TC1900009297.hg.1	TBXA2R	Thromboxane A2 receptor	1.72	0.0043	NM_001060
TC0300011059.hg.1	GPX1	Glutathione peroxidase 1	1.81	0.0016	NM_000581

**Apoptotic process**					
TC1800008891.hg.1	BCL2	B-cell CLL/lymphoma 2	2.73	0.0012	NM_000633
TC2000008815.hg.1	BCL2L1	BCL2-like 1	1.71	0.0100	NM_001191
TC2200006521.hg.1	BCL2L13	BCL2-like 13 (apoptosis facilitator)	1.83	0.0076	NM_001270726
TC0200010972.hg.1	MFF	Mitochondrial fission factor	1.78	0.0012	NM_001277061
TC1900011729.hg.1	TIMM50	Translocase of inner mitochondrial membrane 50 homolog	2.12	<0.0001	NM_001001563
TC1100009101.hg.1	ZBTB16	Zinc finger and BTB domain containing 16	2.04	0.0033	NM_001018011

**Immune response**					
TC0800006746.hg.1	BLK	BLK proto-oncogene, Src family tyrosine kinase	2.60	0.0060	NM_001715
TC1900008166.hg.1	CD79A	CD79a molecule	3.45	0.0008	NM_001783
TC0600011491.hg.1	HLA-DRB5	Major histocompatibility complex, class II, DR β 5	44.50	0.0022	NM_002125
TC1400010492.hg.1	IGHV4-28	Immunoglobulin heavy variable 4-28	14.36	0.0076	AM233776.1
TC1400010806.hg.1	IGHV5-51	Immunoglobulin heavy variable 5-51	9.27	0.0001	FN550293.1
TS00000553.hg.1	IGKV1-27	Immunoglobulin kappa variable 1-27	12.72	0.0016	DQ101172.1
TC0200008384.hg.1	IGKV2D-29	Immunoglobulin kappa variable 2D-29	4.21	0.0030	DQ101153.1
TC2200006771.hg.1	IGLV1-50	Immunoglobulin lambda variable 1-50	4.80	0.0150	FM206563.1
TC1600007312.hg.1	IL4R	Interleukin 4 receptor	3.02	<0.0001	NM_000418
TC1600011368.hg.1	LAT	Linker for activation of T-cells	2.50	0.0001	NM_001014987
TC2200009231.hg.1	MIF	Macrophage migration inhibitory factor	2.27	0.0005	NM_002415
TC1100007771.hg.1	MS4A1	Membrane-spanning 4-domains, subfamily A, 1	2.33	0.0080	NM_021950
TC0300007067.hg.1	MYD88	Myeloid differentiation primary response 88	2.20	0.0008	NM_001172566
TC2200008183.hg.1	VPREB3	Pre-B lymphocyte 3	2.42	0.0090	NM_013378
TC0300013859.hg.1	CD200	CD200 molecule	4.02	<0.0001	NM_001004196

**Angiogenesis**					
TC1500010429.hg.1	CIB1	Calcium and integrin binding 1 (calmyrin)	2.56	<0.0001	NM_001277764
TC1700012274.hg.1	ITGB3	Integrin beta 3	4.29	0.0030	NM_000212
TC0700009977.hg.1	PDGFA	Platelet-derived growth factor alpha polypeptide	1.70	0.0041	NM_002607
TC1700008661.hg.1	PRKCA	Protein kinase C, alpha	1.73	0.0012	NM_002737

**Endothelial cell activation**					
TC0400011053.hg.1	CXCL10	Chemokine (C-X-C motif) ligand 10	5.24	0.0050	NM_001565
TC0300011485.hg.1	FOXP1	Forkhead box P1	1.61	0.0070	NM_001012505
TC1400008684.hg.1	PRMT5	Protein arginine methyltransferase 5	1.78	<0.0001	NM_001039619
TC0100016357.hg.1	SELP	Selectin P	2.66	0.0039	NM_003005

**Cell adhesion**					
TC1100006494.hg.1	CD151	CD151 molecule	1.52	0.0010	NM_001039490
TC1700011435.hg.1	ICAM2	Intercellular adhesion molecule 2	1.56	0.0046	NM_000873
TC1300009165.hg.1	PCDH9	Protocadherin 9	1.62	0.0028	NM_020403
TC1700012274.hg.1	ITGB3	Integrin beta 3	4.29	0.0024	NM_000212
TC1200010794.hg.1	ITGB7	Integrin beta 7	2.10	0.0050	NM_000889

**Extracellular matrix organization**					
TC0100010863.hg.1	LAMC1	Laminin, gamma 1 (formerly LAMB2)	2.06	0.0074	NM_002293
TC0600008462.hg.1	COL19A1	Collagen, type XIX, alpha 1	2.94	0.0012	NM_001858
TC2000008678.hg.1	CST3	Cystatin C	2.27	0.0030	NM_000099
TC0600006873.hg.1	BMP6	Bone morphogenetic protein 6	2.83	0.0023	NM_001718
TC0500012519.hg.1	SPARC	Secreted protein, acidic, cysteine-rich	3.56	0.0012	NM_001309443

**Cell proliferation**					
TC0800008845.hg.1	MYC	MYC proto-oncogene, bHLH transcription factor	2.05	0.0065	NM_002467
TC1900010696.hg.1	AKT2	v-Akt murine thymoma viral oncogene homolog 2	2.36	<0.0001	NM_001243027
TC0600007862.hg.1	PIM1	Pim-1 proto-oncogene, serine/threonine kinase	2.15	0.0122	NM_001243186
TC0200015887.hg.1	CUL3	Cullin 3	-1.54	0.0008	NM_001257197
TC0600011809.hg.1	CCND3	Cyclin D3	1.93	<0.0001	NM_001136017
TC1400010085.hg.1	TCL1A	T-cell leukemia/lymphoma 1 A	6.87	<0.0001	NM_001098725

**Growth factor and growth factor binding**					
TC0700009977.hg.1	PDGFA	Platelet-derived growth factor alpha polypeptide	1.66	0.0013	NM_002607
TC0300007067.hg.1	MYD88	Myeloid differentiation primary response 88	2.04	0.0004	NM_001172566
TC0600006873.hg.1	BMP6	Bone morphogenetic protein 6	2.92	0.0041	NM_001718

**Type I interferon signaling pathway**					
TC0100015921.hg.1	ADAR	Adenosine deaminase, RNA-specific	1.84	0.0001	NM_001025107
TC0600007530.hg.1	HLA-E	Major histocompatibility complex, class I, E	1.72	0.0003	NM_005516
TC0600007487.hg.1	HLA-G	Major histocompatibility complex, class I, G	1.94	0.0015	NM_002127
TC0100013445.hg.1	IFI6	Interferon, alpha-inducible protein 6	1.83	0.0066	NM_002038
TC1400010584.hg.1	IRF9	Interferon regulatory factor 9	1.82	0.0031	NM_006084
TC1500008232.hg.1	ISG20	Interferon stimulated exonuclease gene 20 kDa	2.52	0.0001	NM_001303233
TC0300007067.hg.1	MYD88	Myeloid differentiation primary response 88	2.13	0.0006	NM_001172566
TC1200012708.hg.1	OAS1	2-5-Oligoadenylate synthetase 1	2.46	0.0108	NM_001032409
TC1200010908.hg.1	STAT2	Signal transducer and activator of transcription 2	1.58	0.0028	NM_005419
TC0400011053.hg.1	CXCL10	Chemokine (C-X-C motif) ligand 10	5.23	0.0040	NM_001565
TC1400010721.hg.1	PSME2	Proteasome activator subunit 2; microRNA 7703	1.70	0.0010	NM_002818

**Epidermal growth factor receptor signaling pathway**					
TC1900010696.hg.1	AKT2	v-Akt murine thymoma viral oncogene homolog 2	2.37	<0.0001	NM_001243027
TC1900006864.hg.1	MAP2K7	Mitogen-activated protein kinase kinase 7	2.24	0.0041	NM_001297555
TC0200008742.hg.1	NCK2	NCK adaptor protein 2	2.54	<0.0001	NM_001004720
TC1200009071.hg.1	PEBP1	Phosphatidylethanolamine binding protein 1	1.68	0.0007	NM_002567

**Transforming growth factor-beta signaling pathway**					
TC0600006873.hg.1	BMP6	Bone morphogenetic protein 6	2.91	0.0023	NM_001718
TC0600013126.hg.1	PTPRK	Protein tyrosine phosphatase, receptor type, K	3.07	0.0033	NM_001291983
TC1600009200.hg.1	TRAP1	TNF receptor-associated protein 1	1.96	0.0050	NM_001272049
TC1900011636.hg.1	UBE2M	Ubiquitin-conjugating enzyme E2M	1.73	0.0046	NM_003969

**Interleukin signaling pathway**					
TC1000009691.hg.1	IL2RA	Interleukin 2 receptor, alpha	2.46	0.0010	NM_000417
TC1600007312.hg.1	IL4R	Interleukin 4 receptor	2.94	<0.0001	NM_000418
TC0800008845.hg.1	MYC	MYC proto-oncogene, bHLH transcription factor	2.13	0.0065	NM_002467
TC0 × 00010196.hg.1	RPS6KA6	Ribosomal protein S6 kinase, 90 kDa, polypeptide 6	2.02	0.0012	NM_014496
TC1200010908.hg.1	STAT2	Signal transducer and activator of transcription 2	1.54	0.0022	NM_005419

**Wnt signaling pathway**					
TC1900009240.hg.1	GNG7	Guanine nucleotide binding protein, gamma 7	2.42	0.0010	NM_052847
TC0800008845.hg.1	MYC	MYC proto-oncogene, bHLH transcription factor	2.16	0.0066	NM_002467
TC1800007805.hg.1	NFATC1	Nuclear factor of activated T-cells, 1	2.02	0.0042	NM_001278669
TC0300012807.hg.1	SIAH2	Siah E3 ubiquitin protein ligase 2	1.94	0.0011	NM_005067
TC1900009176.hg.1	TCF3	Transcription factor 3	1.93	0.0032	NM_001136139
TC0900010543.hg.1	TLE1	Transducin-like enhancer of split 1 [E(sp1) homolog]	2.13	0.0012	NM_001303103

**Glycolysis**					
TC1000007891.hg.1	HK1	Hexokinase 1	1.91	0.0011	NM_000188
TC2100007355.hg.1	PFKL	Phosphofructokinase, liver	1.86	0.0030	NM_001002021
TC1500009952.hg.1	PKM	Pyruvate kinase, muscle	1.63	0.005	NM_001206796
TC1200006649.hg.1	TPI1	Triosephosphate isomerase 1	1.54	0.0029	NM_000365

**Platelet derived growth factor (PDGF) signaling pathway**					
TC1900010696.hg.1	AKT2	v-Akt murine thymoma viral oncogene homolog 2	2.34	<0.0001	NM_001243027
TC0 × 00009069.hg.1	ARHGAP6	Rho GTPase activating protein 6	2.54	0.0052	NM_001287242
TC0800008845.hg.1	MYC	MYC proto-oncogene, bHLH transcription factor	2.05	0.0080	NM_002467
TC0200008742.hg.1	NCK2	NCK adaptor protein 2	2.53	<0.0001	NM_001004720
TC0700009977.hg.1	PDGFA	Platelet-derived growth factor alpha polypeptide	1.52	0.0027	NM_002607

**FAS signaling pathway**					
TC1900010696.hg.1	AKT2	v-Akt murine thymoma viral oncogene homolog 2	2.32	<0.0001	NM_001243027
TC0800009239.hg.1	CYC1	Cytochrome c-1	1.81	0.0066	NM_001916
TC0900012167.hg.1	GSN	Gelsolin	1.66	0.0041	NM_000177
TC0100017528.hg.1	PARP1	Poly(ADP-ribose) polymerase 1	1.72	0.0012	NM_001618
TC0300007454.hg.1	PARP3	Poly(ADP-ribose) polymerase family member 3	2.05	0.0010	NM_001003931

**Phosphoinositide 3 kinase signaling pathway**					
TC1900010696.hg.1	AKT2	v-Akt murine thymoma viral oncogene homolog 2	2.40	<0.0001	NM_001243027
TC1300008688.hg.1	FOXO1	Forkhead box O1	2.03	0.0008	NM_002015
TC1500009452.hg.1	GNB5	Guanine nucleotide binding protein (G protein), β5	1.65	0.0023	NM_006578
TC1100008136.hg.1	RPS6KB2	Ribosomal protein S6 kinase, 70 kDa, polypeptide 2	1.72	0.0010	NM_003952

Among genes involved in apoptosis, we found the upregulation of B-cell lymphoma 2 (BCL2); BCL2-like 1 (BCL2L1); BCL2-like 13 (BCL2L13); mitochondrial fission factor (MFF); translocase of inner mitochondrial membrane 50 homolog (TIMM50), and zinc finger and BTB domain containing 16 (ZBTB16). Interestingly, TIMM50 is an anti-apoptotic gene that has been involved in breast cancer cell proliferation (26).

The upregulation of apoptotic molecules was paralleled by the overexpression of genes involved in cell proliferation including MYC proto-oncogene, bHLH transcription factor (MYC); v-akt murine thymoma viral oncogene homolog 2 (AKT2); Pim-1 proto-oncogene, serine/threonine kinase (PIM); cyclin D3 (CCND3), and T-cell leukemia/lymphoma 1 A (TCL1A).

Interestingly, all these molecules have been previously associated to different type of cancer (27–30). Moreover, we found downregulation for cullin 3 (CUL3) a tumor suppressor molecule (31). All these observations may suggest that a dysregulation of proliferative genes in patients with SSc may predispose to the development of malignancies.

We also found upregulation of growth factors encoding molecules such as platelet-derived growth factor alpha polypeptide (PDGFA), bone morphogenetic factor 6 (BM6), and myeloid differentiation primary response 88 (MYD88) a fibrogenic molecule that controls pericyte migration and trans-differentiation of endothelial cells to myofibroblasts (32).

Chronic inflammation is a feature of SSc; therefore, the upregulation of genes involved in the inflammatory response, including chemokine (C-X-C motif) ligand 10 (CXCL10), chemokine (C-X-C motif) receptor 5 (CXCR5), folate receptor 2 (FOLR2), interleukin 2 receptor, alpha (IL2RA), thromboxane A2 receptor (TBXA2R), and glutathione peroxidase 1 (GPX1), is not surprising.

Scleroderma, like other autoimmune connective tissue diseases, is characterized by immune system alterations and the results of our analysis also highlighted this aspect, showing a modulation of a large number of genes involved in immune response. A selection of these molecules is reported in Table 2 and includes: BLK proto-oncogene, Src family tyrosine kinase; CD79a molecule, immunoglobulin-associated alpha (CD79A); major histocompatibility complex, class II, DR beta 5 (HLA-DRB5); immunoglobulin heavy variable 4-28 (IGHV4-28); immunoglobulin heavy variable 5-51 (IGHV5-51); (IGKV1-27); immunoglobulin kappa variable 2D-29; immunoglobulin lambda variable 1-50 (IGLV1-50); interleukin 4 receptor (IL4R), linker for activation of T-cells (LAT); macrophage migration inhibitory factor (MIF); membrane-spanning 4-domains, subfamily A, member 1 (MS4A1); myeloid differentiation primary response 88 (MYD88); pre-B lymphocyte 3 (VPREB3), and CD200 molecule (CD200). Among the abovementioned molecules, MIF has been described as a lung metastasis inducer (33) and CD200 has been involved in the development of breast cancer metastasis (34) and of myeloid leukemia (35).

The initial stages of SSc are accompanied by an angiogenic response to tissue ischemia and vascular damage, that is later replaced by a deficient wound healing and by fibrosis (36). Indeed, several genes involved in angiogenesis were upregulated in lSSc samples including calcium and integrin binding 1 (CIB1); integrin beta 3 (ITGB3), platelet-derived growth factor alpha polypeptide (PDGFA), and protein kinase C, alpha (PRKCA). Interestingly, PDGF stimulates tumor cells, promotes angiogenesis, and the development of cancer associated fibroblasts (37) leading to tumor progression. In addition, aberrant expression of PKCA is associated with a range of malignancies and has recently become a target for anti-cancer therapies (38).

The upregulation of genes such as chemokine (C-X-C motif) ligand 10 (CXCL10), forkhead box P1(FOXP1), protein arginine methyltransferase 5(PRMT5), and selectin P (SELP) is consistent with the endothelial cells activation that is typical of SSc and is accompanied by the overexpression of cell adhesion molecules involved in endothelial cells and leukocytes interactions as well as in blood cells extravasation. We indeed found upregulation for intercellular adhesion molecule 2 (ICAM2), protocadherin 9 (PCDH9), integrin beta 3 (ITGB3), integrin beta 7 (ITGB7), and CD151 molecule that, importantly, is an emerging possible poor prognostic factor for solid tumors (39).

Systemic sclerosis is characterized by connective tissue fibrosis of skin and internal organs that is sustained by extracellular matrix (ECM) remodeling (40). Accordingly, we found an increased expression of transcripts that play a role in ECM organization including laminin gamma 1 (LAMC1), collagen, type XIX (COL19A1), bone morphogenetic protein 6 (BMP6), cystatin C (CST3), and secreted protein, acidic, cysteine-rich (SPARC), a secreted protein that is overexpressed in the fibroblasts of skin biopsy from patients with SSc (41). Noteworthy, remodeling of the stromal ECM by cancer-associated fibroblasts is crucial for tumor cell migration and invasion (42) and SPARC has been associated to these events (43). Interestingly, an elevated preoperative CST3 level was demonstrated to be related with worse survival in patients with renal cell carcinoma (44).

The pathway enrichment analysis that we performed to find signaling network that were overrepresented by modulated genes in lSSc samples, showed an enrichment in apoptosis, glycolysis, PDGF, 5HT4 type receptor, Fas cell surface death receptor (FAS), histamine H2 receptor, serine glycine biosynthesis, beta 3 adrenergic receptor, angiotensin (through G proteins and beta-arrestin), and interleukin signaling pathways (Table 3).

**Table 3:** Pathways enriched in genes modulated in lSSc samples.

Panther pathways	*p*-Value
Apoptosis signaling pathway (P00006)	<0.01
Glycolysis (P00024)	<0.01
Platelet derived growth factor signaling pathway (P00047)	<0.01
5HT4 type receptor mediated signaling pathway (P04376)	0.01
FAS signaling pathway (P00020)	0.01
Histamine H2 receptor mediated signaling pathway (P04386)	0.01
Serine glycine biosynthesis (P02776)	0.02
Beta3 adrenergic receptor signaling pathway (P04379)	0.02
Angiotensin II-stimulated signaling through G proteins and beta-arrestin (P05911)	0.03
Interleukin signaling pathway (P00036)	0.05

Interestingly, several of the abovementioned enriched pathways are involved in cancer development.

Other modulated transcripts belong to the epidermal growth factor (EGF), transforming growth factor (TGF) beta, Wnt, and PI3 kinase signalings, all involved not only in the development of the SSc associated fibrosis (45, 46) but also in cancer development (47–50). In particular, we observed overexpression of the AKT2 member of the EGF signaling pathway and of the FOXO1 component of the PI3K pathway. The deregulation of these genes has been associated with tumor progression and metastatic spread (29, 51).

The functional classification of DEGs in dSSc samples shows that they reflects the gene modulation observed in lSSC samples (see Table 4).

**Table 4:** Selection of modulated genes in diffuse SSc patients versus healthy controls.

ID	Gene symbol	Description	Fold change	*p*-Value	Public gene IDs
**Apoptosis**					
TC1500008231.hg.1	AEN	Apoptosis enhancing nuclease	2.04	0.0024	NM_022767
TC1800008891.hg.1	BCL2	B-cell CLL/lymphoma 2	1.90	0.0090	NM_000633
TC1600009992.hg.1	BCL7C	B-cell CLL/lymphoma 7 C	1.56	0.0101	NM_001286526
TC0200015242.hg.1	STAT1	Signal transducer and activator of transcription 1	2.40	0.0042	NM_007315
TC1900011729.hg.1	TIMM50	Translocase of inner mitochondrial membrane 50	1.76	0.0024	NM_001001563
TC0900009242.hg.1	TRAF2	TNF receptor-associated factor 2	1.52	0.0089	NM_021138

**Cell proliferation**					
TC0300009916.hg.1	HES1	Hes family bHLH transcription factor 1	2.03	<0.0001	NM_005524
TC0900007863.hg.1	CKS2	CDC28 protein kinase regulatory subunit 2	1.50	0.0066	NM_001827
TC0200015887.hg.1	CUL3	Cullin 3	-1.94	0.0068	NM_001257197
TC0600011809.hg.1	CCND3	Cyclin D3	1.56	0.0007	NM_001136017
TC0800008845.hg.1	MYC	MYC proto-oncogene, bHLH transcription factor	2.09	0.0072	NM_002467
TC1900010696.hg.1	AKT2	v-Akt murine thymoma viral oncogene homolog 2	1.96	0.0012	NM_001243027
TC0600007862.hg.1	PIM1	Pim-1 proto-oncogene, serine/threonine kinase	2.12	0.0012	NM_001243186

**Growth factor and growth factor binding**					
TC1900011707.hg.1	GPI	Glucose-6-phosphate isomerase	1.82	0.0016	NM_000175
TC0300007067.hg.1	MYD88	Myeloid differentiation primary response 88	2.50	<0.0001	NM_001172566
TC1100011258.hg.1	FIBP	Fibroblast growth factor intracellular binding protein	1.70	0.0092	NM_004214

**Inflammatory response**					
TC0400011053.hg.1	CXCL10	Chemokine (C-X-C motif) ligand 10	6.69	0.0001	NM_001565
TC0400011054.hg.1	CXCL11	Chemokine (C-X-C motif) ligand 11	2.01	0.0023	NM_001302123
TC1100009225.hg.1	CXCR5	Chemokine (C-X-C motif) receptor 5	3.31	0.0066	NM_001716
TC0 × 00009667.hg.1	FCGR1B	Fc fragment of IgG, high affinity Ib, receptor (CD64)	2.52	0.0076	NM_001004340
TC1400009239.hg.1	FCGR3A	Fc fragment of IgG, low affinity IIIa, receptor (CD16a)	2.00	0.0012	NM_000569
TC1600011368.hg.1	LAT	Linker for activation of T-cells	1.80	0.0042	NM_001014987
TC1900009134.hg.1	PTGER4	Prostaglandin E receptor 4 (subtype EP4)	2.11	0.0020	NM_000958

**Immune response**					
TC1700009612.hg.1	CD79B	CD79b molecule immunoglobulin-associated beta	2.80	0.0020	NM_000626
TC1100011888.hg.1	CR2	Complement component receptor 2	1.90	0.0015	NM_001006658
TC1900011707.hg.1	FOXP3	Forkhead box P3	2.01	0.0066	NM_001114377
TC0600007530.hg.1	HLA-A	Major histocompatibility complex, class I, A	2.11	0.0002	NM_001242758
TC0600007487.hg.1	HLA-B	Major histocompatibility complex, class I, B	1.54	0.0022	D83043.1
TC1700012468.hg.1	HLA-E	Major histocompatibility complex, class I, E	1.66	0.0034	NM_005516
TC1700007931.hg.1	HLA-G	Major histocompatibility complex, class I, G	1.52	0.0045	NM_002127
TC1100008330.hg.1	IGHV3-66	Immunoglobulin heavy variable 3-66	3.50	0.0060	EU667609.1
TC1600007312.hg.1	IGHV4-61	Immunoglobulin heavy variable 4-61	3.51	0.0027	FN550331.1
TC1400010584.hg.1	IL4R	Interleukin 4 receptor	2.02	<0.0001	NM_000418
TC1500008668.hg.1	LAT	Linker for activation of T-cells	1.97	0.0011	NM_001014987
TC1100013178.hg.1	LST1	Leukocyte specific transcript 1	2.11	0.0052	NM_007161
TC1200008920.hg.1	MYD88	Myeloid differentiation primary response 88	2.09	0.0006	NM_001172566
TC1900011255.hg.1	RSAD2	Radical S-adenosyl methionine domain containing 2	8.34	0.0001	NM_080657
TC1000008236.hg.1	TAP1	Transporter 1, ATP-binding cassette, sub-family B	2.00	0.0017	NM_001292022

**Angiogenesis**					
TC1500010429.hg.1	CIB1	Calcium and integrin binding 1 (calmyrin)	2.08	0.0008	NM_001277764
TC1900011707.hg.1	GPI	Glucose-6-phosphate isomerase	1.50	0.0022	NM_000175
TC1700012468.hg.1	HN1	Hematological and neurological expressed 1	2.15	0.0001	NM_001002032
TC0700011770.hg.1	KRIT1	KRIT1, ankyrin repeat containing	−2.03	0.0054	NM_001013406

**Endothelial cell activation**					
TC1100009225.hg.1	CXCL10	Chemokine (C-X-C motif) ligand 10	5.94	0.0004	NM_001565
TC0600006967.hg.1	EDN1	Endothelin 1	2.23	0.0042	NM_001168319

**Extracellular matrix organization**					
TC1900006470.hg.1	BSG	Basigin	2.05	0.0022	NM_001728
TC2000008678.hg.1	CST3	Cystatin C	2.13	0.0014	NM_000099
TC0800010204.hg.1	ADAM2	ADAM metallopeptidase domain 2	1.56	0.0084	NM_001278114

**Type I interferon signaling pathway**					
TC0100015921.hg.1	ADAR	Adenosine deaminase, RNA-specific	1.94	0.0006	NM_001025107
TC1700007931.hg.1	IFI35	Interferon-induced protein 35	1.70	0.0053	NM_005533
TC0100013445.hg.1	IFI6	Interferon, alpha-inducible protein 6	4.00	<0.0001	NM_002038
TC1000008400.hg.1	IFIT1	Interferon-induced protein with tetratricopeptide repeats 1	7.03	0.0030	NM_001270927
TC1000008396.hg.1	IFIT2	Interferon-induced protein with tetratricopeptide repeats 2	13.27	<0.0001	NM_001547
TC1000008397.hg.1	IFIT3	Interferon-induced protein with tetratricopeptide repeats 3	18.12	0.0010	NM_001031683
TC1100009657.hg.1	IFITM3	Interferon induced transmembrane protein 3	2.11	0.0008	NM_021034
TC0500012017.hg.1	IRF1	Interferon regulatory factor 1	1.73	0.0063	NM_002198
TC1400010584.hg.1	IRF9	Interferon regulatory factor 9	1.80	0.0007	NM_006084
TC0100006483.hg.1	ISG15	ISG15 ubiquitin-like modifier	2.36	0.0011	NM_005101
TC1500008232.hg.1	ISG20	Interferon stimulated exonuclease gene 20 kDa	2.00	0.0021	NM_001303233
TC0300007067.hg.1	MYD88	Myeloid differentiation primary response 88	2.27	0.0001	NM_001172566
TC1200012708.hg.1	OAS1	2-5-oligoadenylate synthetase 1	3.95	0.0004	NM_001032409
TC0200016402.hg.1	RSAD2	Radical S-adenosyl methionine domain containing 2	8.23	0.0003	NM_080657

**Ras protein signal transduction**					
TC0700007367.hg.1	DBNL	Drebrin-like	2.02	0.0027	NM_001014436
TC0300007432.hg.1	MAPKAPK3	Mitogen-activated protein kinase(PK)-activated PK 3	1.94	0.0031	NM_001243925
TC1600011368.hg.1	LAT	Linker for activation of T-cells	1.76	0.0020	NM_001014987
TC2200008641.hg.1	RAC2	Rho family, small GTP binding protein Rac2	1.88	0.0066	NM_002872
TC0100018463.hg.1	RHOC	Ras homolog family member C	2.59	0.0041	NM_001042678

**p53 pathway**					
TC2100008385.hg.1	SUMO3	Small ubiquitin-like modifier 3	2.44	0.0031	NM_001286416
TC1900007688.hg.1	CCNE1	Cyclin E1	1.70	0.0066	NM_001238
TC0900009242.hg.1	TRAF2	TNF receptor-associated factor 2	1.73	0.0043	NM_021138
TC1900010696.hg.1	AKT2	v-akt murine thymoma viral oncogene homolog 2	1.77	0.0002	NM_001243027
TC1900007688.hg.1	CCNE1	Cyclin E1	1.83	0.0027	NM_001238

**JAK-STAT cascade**					
TC0300007256.hg.1	CCR2	Chemokine (C-C motif) receptor 2	−3.18	0.0080	NM_001123041
TC0300009916.hg.1	HES1	Hes family bHLH transcription factor 1	2.08	0.0011	NM_005524
TC1200010908.hg.1	STAT2	Signal transducer and activator of transcription 2	2.04	0.0010	NM_005419
TC0200015242.hg.1	STAT1	Signal transducer and activator of transcription 1	2.56	0.0034	NM_007315

**Wnt signaling pathway**					
TC1400006718.hg.1	PSME1	Proteasome activator subunit 1	2.00	0.0013	NM_001281528
TC0600014110.hg.1	PSMB9	Proteasome subunit beta 9	2.40	0.0001	NM_002800
TC1400010721.hg.1	PSME2	Proteasome activator subunit 2; microRNA 7703	2.66	0.0001	NM_002818
TC1400007320.hg.1	DACT1	Disheveled-binding antagonist of beta-catenin 1	4.54	0.0007	NM_001079520
TC0800008845.hg.1	MYC	MYC proto-oncogene, bHLH transcription factor	2.61	0.0076	NM_002467
TC1000008942.hg.1	TCF7L2	Transcription factor 7-like 2 (T-cell specific, HMG-box)	1.76	0.0081	NM_001146274

**Glycolysis**					
TC1900011707.hg.1	GPI	Glucose-6-phosphate isomerase	1.60	0.0015	NM_000175
TC1200006649.hg.1	TPI1	Triosephosphate isomerase 1	2.00	0.0005	NM_000365
TC2100007355.hg.1	PFKL	Phosphofructokinase, liver	2.01	<0.0001	NM_001002021

**Platelet derived growth factor signaling pathway**					
TC1000007761.hg.1	ARID5B	AT rich interactive domain 5B (MRF1-like)	2.50	0.0033	NM_001244638
TC0800008845.hg.1	MYC	MYC proto-oncogene, bHLH transcription factor	2.43	0.0086	NM_002467
TC0300007432.hg.1	MAPKAPK3	Mitogen-activated protein kinase(PK)-activated PK 3	2.05	0.0032	NM_001243925
TC1900010696.hg.1	AKT2	v-Akt murine thymoma viral oncogene homolog 2	1.97	0.0001	NM_001243027

**Interleukin signaling pathway**					
TC1200010908.hg.1	STAT2	Signal transducer and activator of transcription 2	1.54	0.0002	NM_005419
TC0200015242.hg.1	STAT1	Signal transducer and activator of transcription 1	2.27	0.0012	NM_007315
TC0800008845.hg.1	MYC	MYC proto-oncogene, bHLH transcription factor	2.12	0.0078	NM_002467
TC1600007312.hg.1	IL4R	Interleukin 4 receptor	1.92	0.0001	NM_000418

**p38 signaling pathway**					
TC2200008641.hg.1	RAC2	Rho family, small GTP binding protein Rac2	1.71	0.0054	NM_002872
TC1700008757.hg.1	MAP2K6	Mitogen-activated protein kinase kinase 6	−1.66	0.0042	NM_002758
TC0300007432.hg.1	MAPKAPK3	Mitogen-activated protein kinase(PK)-activated PK 3	1.92	0.0077	NM_001243925

**Epidermal growth factor receptor signaling pathway**					
TC0200007446.hg.1	PRKCE	Protein kinase C, epsilon	1.58	0.0020	NM_005400
TC2200008641.hg.1	RAC2	Rho family, small GTP binding protein Rac2	1.82	0.0031	NM_002872
TC1200010908.hg.1	STAT2	Signal transducer and activator of transcription 2	1.80	0.0003	NM_005419
TC0200015242.hg.1	STAT1	Signal transducer and activator of transcription 1	2.53	0.0042	NM_007315
TC1900010696.hg.1	AKT2	v-Akt murine thymoma viral oncogene homolog 2	2.06	0.0006	NM_001243027

The apoptosis functional class accounted for several upregulated transcripts namely apoptosis enhancing nuclease (AEN), B-cell CLL/lymphoma 2 (BCL2), B-cell CLL/lymphoma 7 C (BCL7C), signal transducer and activator of transcription 1 (STAT1), TNF receptor-associated factor 2 (TRAF2), and translocase of inner mitochondrial membrane 50 homolog (TIMM50). In addition to genes that were also modulated in lSSc samples (i.e., MYC, AKT2, and PIM1), other genes involved in cell proliferation and also in tumor development were overexpressed. These genes include: hes family bHLH transcription factor 1(HES1), CDC28 protein kinase regulatory subunit 2 (CKS2), and cyclin D3 (CCND3) (52–54). Moreover, three upregulated transcripts were ascribed to the growth factor binding gene category: glucose-6-phosphate isomerase (GPI), fibroblast growth factor intracellular binding protein (FIBP), and MYD88 (also modulated in lSSc samples as mentioned above). Noteworthy, also in dSSc samples, we observed the down-modulation of the tumor suppressor CUL3.

Several modulated genes encode for inflammatory molecules including chemokine (C-X-C motif) ligand 11 (CXCL11), Fc fragment of IgG, high affinity Ib, receptor (FCGR1B), Fc fragment of IgG, low affinity IIIa, receptor (FCGR3A), linker for activation of T-cells (LAT), prostaglandin E receptor 4 (PTGER4), CXCL10, and CXCR5. These last two transcripts were also overexpressed in lSSc samples.

Genes involved in the immune response were modulated also in dSSc samples and, besides the genes overexpressed in lSSc samples (i.e., IL4R, LAT, and MYD88), we found upregulation of CD79b molecule, immunoglobulin-associated beta (CD79B), complement component receptor 2 (CR2), forkhead box P3 (FOXP3), major histocompatibility complex, class I, A (HLA-A), major histocompatibility complex, class I, B (HLA-B), major histocompatibility complex, class I, E (HLA-E), major histocompatibility complex, class I, G (HLA-G), immunoglobulin heavy variable 3-66 (IGHV3-66), immunoglobulin heavy variable 4-61 (IGHV4-61), radical S-adenosyl methionine domain containing 2 (RSAD2), and transporter 1, ATP-binding cassette, sub-family B (TAP1).

Among DEGs involved in angiogenesis there are: glucose-6-phosphate isomerase (GPI), hematological and neurological expressed 1 (HN1), KRIT1, ankyrin repeat containing (KRIT1) and CIB1 (also modulated in lSSc samples).

Endothelial activation was well represented in dSSc samples by the overexpression of gene encoding for endothelin 1 (EDN1) that is associated with diseases characterized by endothelial dysfunction and fibrosis (55).

Finally, we found upregulation of three transcripts that play a role in the ECM organization including ADAM metallopeptidase domain 2 (ADAM2), CST3 (increased also in lSSc samples), and basigin (BSG). BSG, also named ECM metalloproteinase inducer (EMMPRIN), is expressed on the surface of tumor cells and induces fibroblasts to synthesize matrix metalloproteinases (56).

Pathways enrichment analysis showed that pathways also overrepresented in lSSc samples were enriched in dSSc samples (i.e., glycolysis, apoptosis, and interleukin signaling pathway). Besides these signaling pathways, we found an enrichment in pathways involved in tumor development including: inflammation mediated by chemokine and cytokine, cell cycle, Ras (57), oxidative stress response (58), p53 (59), ubiquitin proteasome (60), JAK/STAT (61), p38 MAPK (62), angiogenesis, and EGF receptor signaling pathway (Table 5). Among modulated genes belonging to these pathways, we mention ras homolog family member C (RHOC), the overexpression of which indicates poor prognosis in breast cancer cells (63), and the abovementioned AKT2 and HES1.

**Table 5:** Pathways enriched in genes modulated in diffuse SSc samples.

Panther pathways	*p*-Value
Glycolysis (P00024)	0.005
Apoptosis signaling pathway (P00006)	0.006
Inflammation mediated by chemokine and cytokine signaling pathway (P00031)	0.008
Cell cycle (P00013)	0.009
Ras pathway (P04393)	0.010
Oxidative stress response (P00046)	0.012
p53 pathway (P00059)	0.018
Ubiquitin proteasome pathway (P00060)	0.020
Interleukin signaling pathway (P00036)	0.026
JAK/STAT signaling pathway (P00038)	0.030
p38 MAPK pathway (P05918)	0.032
Angiogenesis (P00005)	0.035
Epidermal growth factor receptor signaling pathway (P00018)	0.044

As found in lSSc samples, among other DEGs involved in signal transduction, several transcripts were involved in Wnt, PDGF, and type I interferon signaling pathway and, their role in cancer development has been already stressed in our dissection of genes modulated in lSSc. Not surprisingly, type I interferon pathway accounted for a large amount of DEGs (14) and the evidence of the modulation of this molecular signaling both in lSSc and in dSSc further underlines its role in the pathogenesis of the disease.

### Network Analysis of Modulated Genes in SSc

The gene expression profiling of SSc patients was complemented with a network analysis. With this purpose, by a bioinformatic analysis, we selected all the functional and experimentally validated interactions between the protein products of modulated genes and we constructed the two protein–protein interaction (PPI) networks that were representative of lSSc and dSSc dataset.

**Figure 1:**
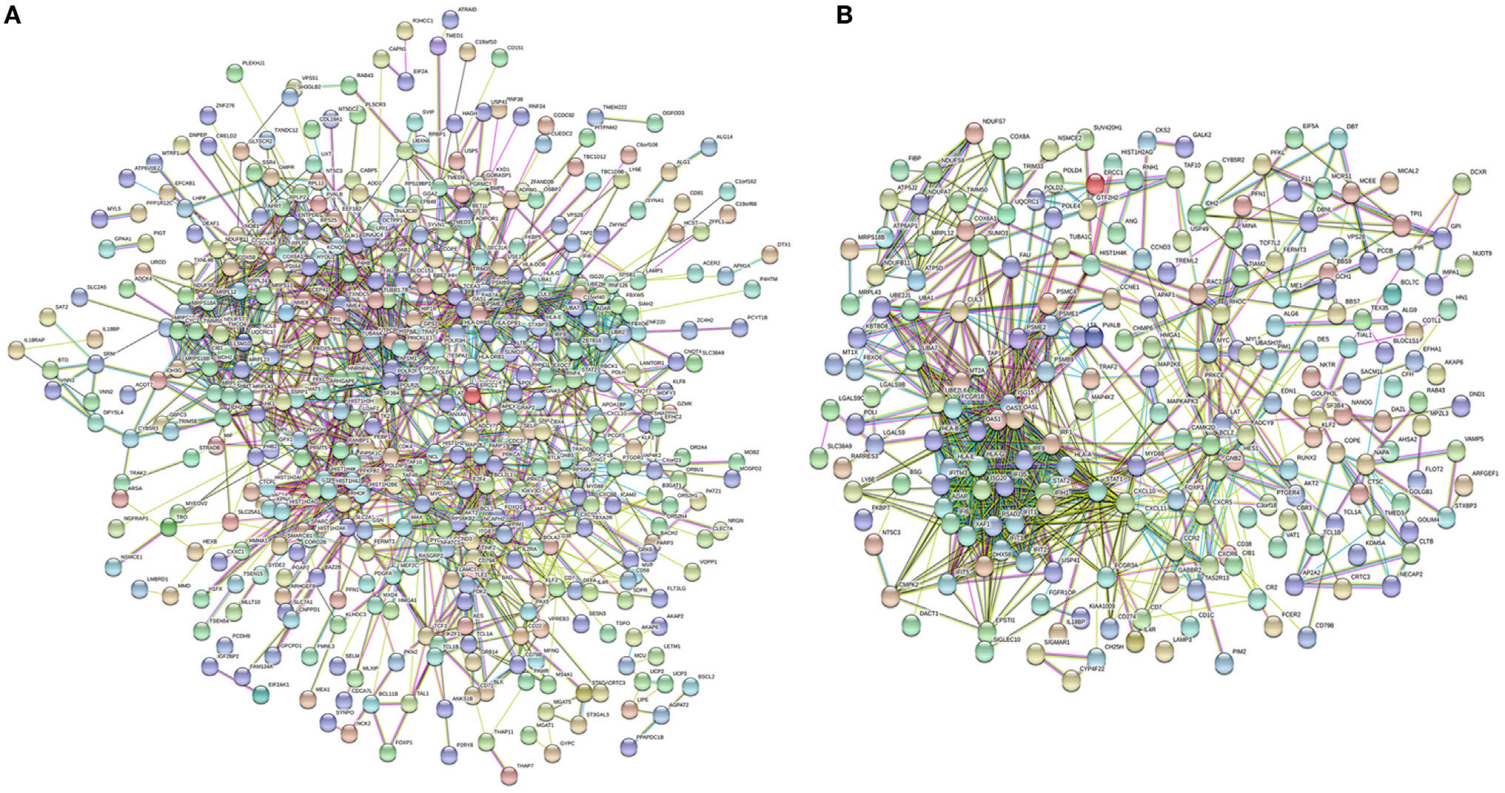
Network analysis of modulated genes in systemic sclerosis (SSc) patients. Protein–protein interaction network of differently expressed genes in lSSc **(A)** and in diffuse cutaneous SSc **(B)** samples.

The lSSc-PPI network comprised 440 genes (nodes) and 1351 pairs of interactions (edges) (Figure 1A), whereas the dSSc included 225 genes and 870 pairs of interactions (Figure 1B).

The PPI networks were then submitted to a modular analysis to find set of highly interconnected nodes (modules) that participate in multiple activities in a coordinated manner and that are expected to play a prominent role in the development of biological phenomena.

**Figure 2:**
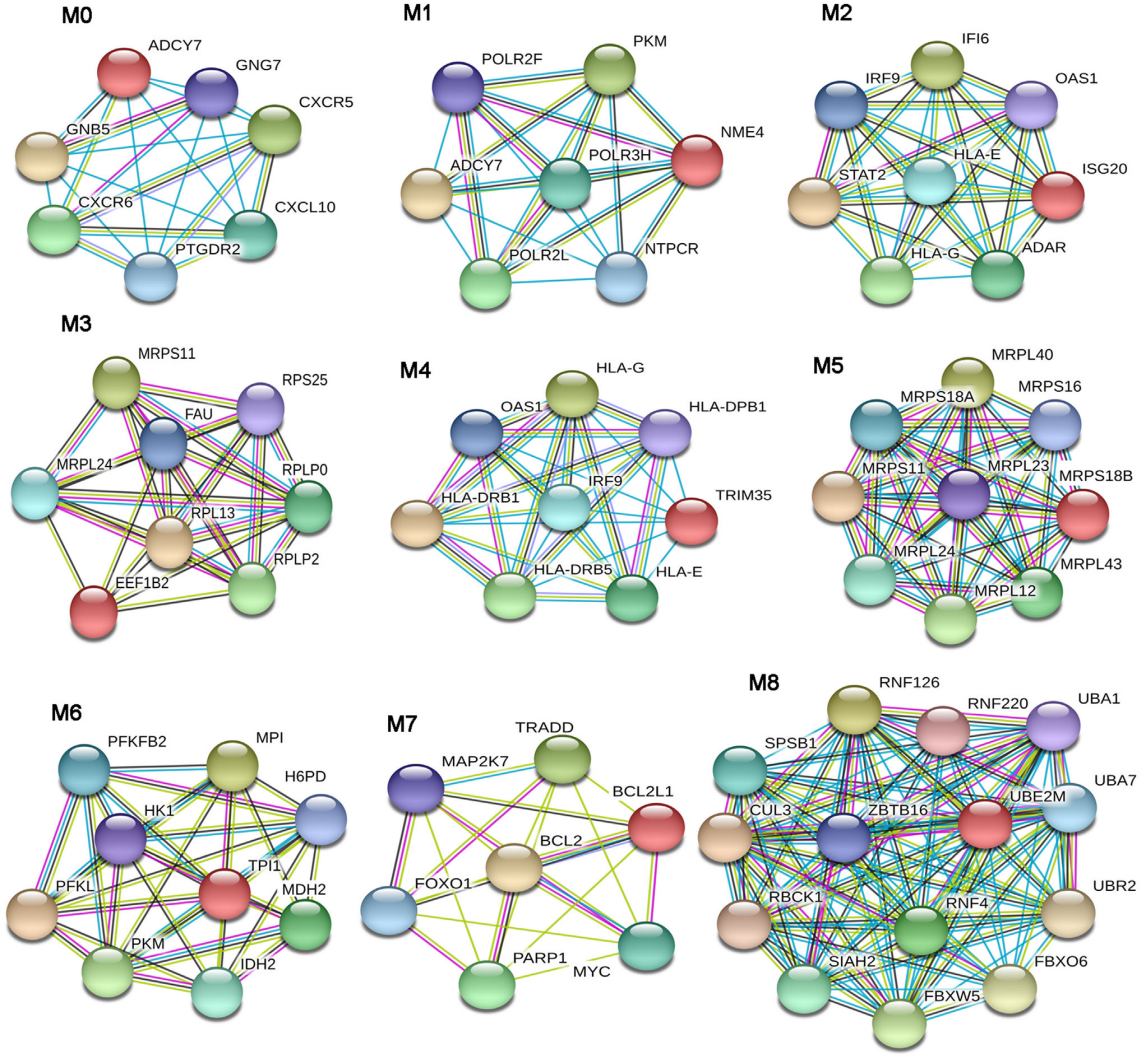
Modular analysis of the lSSc-protein–protein interaction network. Modules originated from the interaction network of modulated genes in lSSc samples.

In the lSSc-PPI network, we identified nine modules that are graphically represented in Figure 2. Moreover, a functional enrichment analysis was applied to find the associations between each module and relevant enriched “GO terms” and pathways. All the significantly enriched (*p* < 0.05) biological processes (BPs) and pathways in each module are showed in Table S3 in Supplementary Material and Table 6 shows a selection of the most relevant terms. We observed that the most enriched BPs in module M0, were the signaling of G-protein coupled receptors (GPCRs) and mediated by chemokine. Interestingly, the GPCRs pathway is involved in cancer initiation and progression and GPCRs are emerging as anti-cancer drug targets (64). In the same module M0, we could highlight, among other, an enrichment in inflammation mediated by chemokine and cytokine, PI3 kinase and endothelin signaling pathway. Module M1 showed an enrichment in the positive regulation of type I interferon production BP whereas, among different enriched pathways, we found the glycolysis pathway and again, the endothelin signaling pathway. Module M2 and M4 were the most representative of the type I interferon response. In M2, the GO terms associated to the type I interferon signaling were the most enriched biological processes, followed by terms associated to the innate immune response (i.e., innate immune response, defense response to virus, interferon gamma mediate signaling) and to the adaptive immune response (i.e., antigen processing and presentation of exogenous peptides). Interestingly, the enriched pathways in M2 were related to the Jak/Stat and Interleukin signaling, both associated to tumor development as previously remarked.

**Table 6:** Most relevant biological processes and pathways enriched in lSSc modules.

Gene ontology biological processes	*p* Value	Panther pathways	*p* Value
**M0**			
G-protein coupled receptor signaling pathway	<0.001	inflammation mediated by chemokine and cytokine signaling pathway	<0.001
Chemokine-mediated signaling pathway	0.010	PI3 kinase pathway	0.013
		Endothelin signaling pathway	0.020

**M1**			
Positive regulation of type I interferon production	0.001	Glycolysis	0.010
		Endothelin signaling pathway	0.016

**M2**			
Type I interferon signaling pathway	<0.001	JAK/STAT signaling pathway	0.001
Cellular response to type I interferon	<0.001	Interleukin signaling pathway	0.026
Response to type I interferon	<0.001		
Cytokine-mediated signaling pathway	<0.001		
Innate immune response	<0.001		
Immune response	<0.001		
Defense response to virus	<0.001		
Interferon-gamma-mediated signaling pathway	<0.001		
Antigen processing and presentation of exogenous peptide *via* MHC class I	0.030		

**M3**			
Translation	<0.001	None	
Protein metabolic process	<0.001		
rRNA metabolic process	0.033		

**M4**			
Interferon-gamma-mediated signaling pathway	<0.001	Interferon gamma signaling	<0.001
Cellular response to interferon-gamma	<0.001	Interferon Signaling	<0.001
Response to interferon-gamma	<0.001	Interferon alpha/beta signaling	<0.001
Type I interferon signaling pathway	<0.001	PD-1 signaling	<0.001
Cellular response to type I interferon	<0.001		
Positive regulation of T cell activation	<0.001		
Response to type I interferon	<0.001		
Regulation of T cell activation	<0.001		
Regulation of T cell proliferation	0.001		
Regulation of lymphocyte activation	0.027		
Antigen processing and presentation of exogenous peptide *via* MHC class I	0.032		
Antigen processing and presentation of exogenous peptide *via* MHC class II	0.050		

**M5**			
Translation	0.008	None	

**M6**			
Glycolysis	<0.001	Glycolysis	<0.001
Generation of precursor metabolites and energy	<0.001	Pyruvate metabolism	<0.001
Monosaccharide metabolic process	0.007		
Carbohydrate metabolic process	0.009		

**M7**			
Apoptotic process	<0.001	Apoptosis signaling pathway	<0.001
Programmed cell death	<0.001	FAS signaling pathway	0.010
Regulation of apoptotic process	<0.001	p53 pathway feedback loops 2	0.013
Positive regulation of apoptotic process	0.008	Phosphoinositide 3 kinase pathway	0.016
Negative regulation of apoptotic process	0.027	Ras Pathway	0.030
Extrinsic apoptotic signaling pathway	0.033	Interleukin signaling pathway	0.035
		FGF signaling pathway	0.042
		Epidermal growth factor receptor signaling pathway	0.042

**M8**			
Protein ubiquitination	<0.001	Ubiquitin proteasome pathway	0.001

In M4, the most enriched BPs were interferon-gamma-mediated signaling pathway and cellular response to interferon-gamma. Besides the BPs associated to type I interferon response, we found an enrichment in terms referred to the lymphocyte-associated immune response (i.e., regulation of lymphocyte activation, regulation of T cell activation, regulation of T cell proliferation and antigen processing, and presentation of peptide antigen *via* MHC class I and II). Most enriched pathways in M4 were referred to interferons (alpha, beta and gamma) pathways. Interestingly, in M4, we observed an enrichment in the PD-1 signaling, an immune-inhibitory-checkpoint that acts as crucial mediator for the escape phase of cancer immune editing (65). Module M3 and M5 were enriched in terms related to translation and other metabolic processes of proteins and, in module M6 the most enriched BPs and pathways were referred to the glycolytic pathway. In module M7, we observed an enrichment of many BPs associated to positive and negative regulation of apoptosis and concordantly, an enrichment in the apoptotic FAS and p53 pathways signaling. Moreover, in M7, several cancer-associated signaling pathways were included, including PI3 kinase, Ras, Interleukin, FGF, and EGF receptors signaling pathways. Finally, in module M8, the protein ubiquitination BP and the ubiquitin proteasome pathway were the most over-represented. Interestingly, tumor cells have a high dependency on the proteasome for survive and proteasome deregulation is frequently induced by many types of tumors (66).

**Figure 3:**
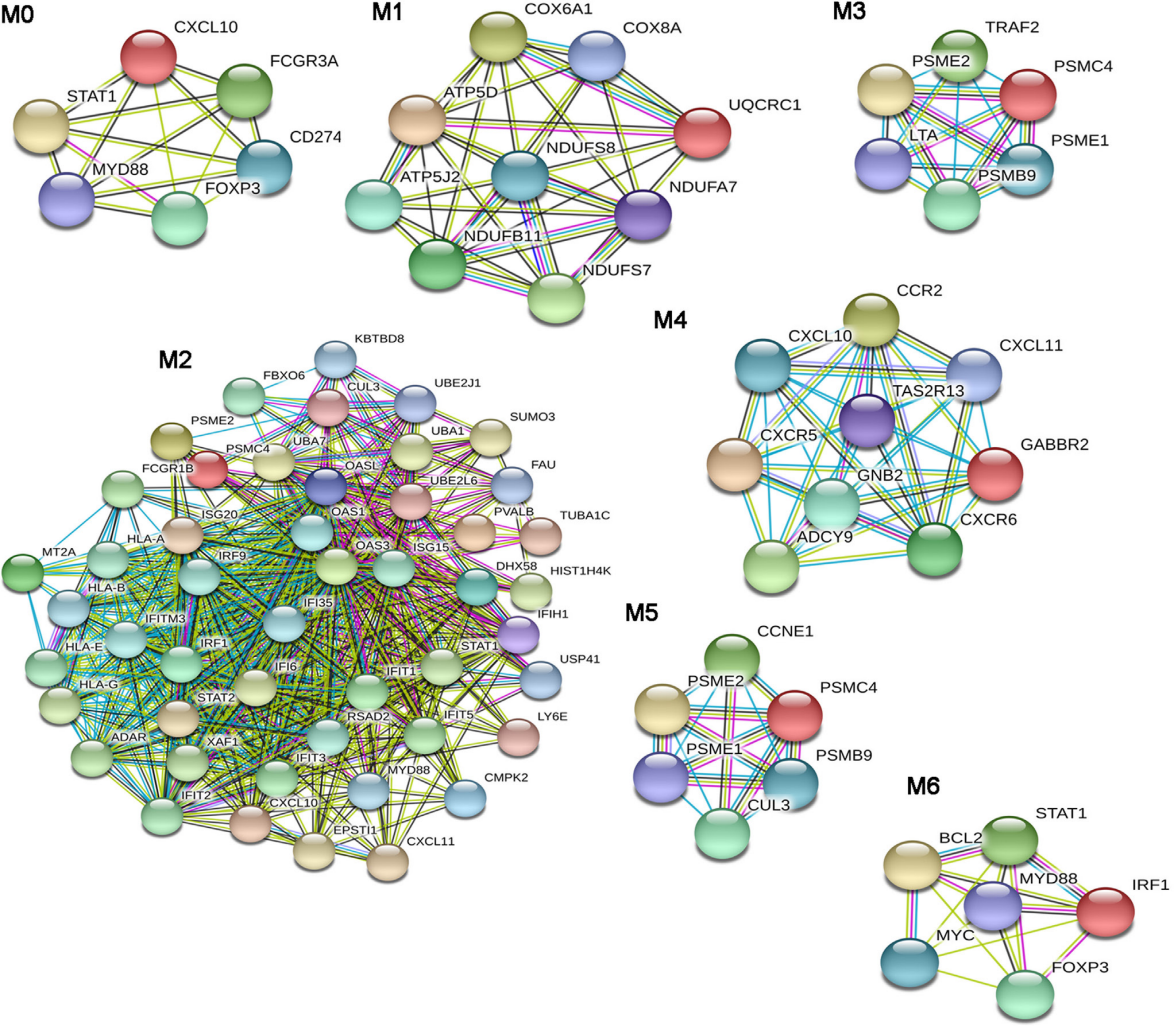
Modular analysis of the diffuse SSc (dSSc)-protein–protein interaction network. Modules originated from the interaction network of modulated genes in dSSc samples.

From the dSSC-PPI network, we could extract seven modules (Figure 3) that were studied by a functional enrichment analysis. The results of this analysis are fully presented in Table S4 in Supplementary Material and the most relevant terms are showed in Table 7.

**Table 7:** Most relevant biological processes and pathways enriched in diffuse SSc modules.

Gene ontology biological processes	*p* Value	Panther pathways	*p* Value
**M0**			
Positive regulation of immune system process	<0.001	Inflammation mediated by chemokine and cytokine signaling pathway	0.001
Regulation of immune system process	0.001	JAK/STAT signaling pathway	0.003
Immune response	0.004	Interferon-gamma signaling pathway	0.010
Regulation of tumor necrosis factor superfamily cytokine production	0.030	p53 pathway feedback loops 2	0.013
Negative regulation of activated T cell proliferation	0.040	Oxidative stress response	0.022
Positive regulation of lymphocyte proliferation	0.050	Toll-like receptor signaling pathway	0.024
Positive regulation of mononuclear cell proliferation	0.050	Ras Pathway	0.026
		Interleukin signaling pathway	0.030
		Epidermal growth factor (EGF) receptor signaling pathway	0.033
		Platelet derived growth factor (PDGF) signaling pathway	0.040
		Angiogenesis	0.050

**M1**			
ATP metabolic process	<0.001	None	
Purine ribonucleoside triphosphate metabolic process	<0.001		
Purine nucleoside triphosphate metabolic process	<0.001		
Purine ribonucleoside monophosphate metabolic process	<0.001		
Purine nucleoside monophosphate metabolic process	<0.001		
Ribonucleoside monophosphate metabolic process	<0.001		
ATP synthesis coupled electron transport	<0.001		

**M2**			
Type I interferon signaling pathway	<0.001	Ubiquitin proteasome pathway	< 0.001
Cellular response to type I interferon	<0.001	JAK/STAT signaling pathway	0.011
Response to type I interferon	<0.001	Interleukin signaling pathway	0.016
Immune system process	<0.001	EGF receptor signaling pathway	0.043
Interferon-gamma-mediated signaling pathway	<0.001	PDGF signaling pathway	0.048
Antigen processing and presentation of exogenous peptide *via* MHC class I	<0.001		
Regulation of immune response	<0.001		

**M3**			
Cellular response to tumor necrosis factor	<0.001	Apoptosis signaling pathway	0.009
Response to tumor necrosis factor	<0.001	Toll-like receptor signaling pathway	0.016
Tumor necrosis factor-mediated signaling pathway	<0.001	Ubiquitin proteasome pathway	0.020
		p53 pathway	0.025

**M4**			
G-protein coupled receptor signaling pathway	<0.001	Inflammation mediated by chemokine and cytokine signaling pathway	<0.001
T cell chemotaxis	<0.001	Heterotrimeric G-protein signaling pathway-Gi alpha and Gs alpha	0.001
Leukocyte chemotaxis	0.012		
Lymphocyte chemotaxis	0.020		

**M5**			
Cell–cell signaling by wnt	<0.001	Cell cycle	<0.001
Wnt signaling pathway	<0.001	p53pathway feedback loops 2	0.010
		Ubiquitin proteasome pathway	0.020
		p53 pathway	0.022

**M6**			
Response to virus	<0.001	Oxidative stress response	<0.001
Regulation of lymphocyte proliferation	0.001	p53 pathway feedback loops 2	<0.001
Alpha-beta T cell differentiation	0.004	Interleukin signaling pathway	<0.001
Alpha-beta T cell activation	0.004	PDGF signaling pathway	0.001
Type I interferon signaling pathway	0.005	JAK/STAT signaling pathway	0.005
Cellular response to type I interferon	0.006	Interferon-gamma signaling pathway	0.009
Response to type I interferon	0.012	Toll-like receptor signaling pathway	0.013
Regulation of type I interferon production	0.027	Ras Pathway	0.020
		Apoptosis signaling pathway	0.028
		EGF receptor signaling pathway	0.028
		Angiogenesis	0.032

The BPs that we found enriched in the module M0 were mainly referred to the immune response. In particular, we observed an enrichment in the regulation of tumor necrosis factor superfamily cytokine production process. Moreover, the over-represented pathways in M0 reassumed nearly all signalings that were enriched in the entire dSSc dataset, including inflammation mediated by chemokine and cytokine, JAK/STAT, p53, oxidative stress response, Ras, interleukin, EGF receptor, and angiogenesis signaling pathway. Module M1 was largely enriched in ATP and purine ribo/nucleoside metabolic processes, whereas M2 was mostly represented by BPs referred to the type I interferon signaling followed by BPs involved in both innate and adaptive immune response. In addition, DEGs in M2 mainly play a role in ubiquitin proteasome, Jak/Stat, interleukin, EGF receptor, and PDGF signaling pathways. In M3, the most enriched BP was cellular response to TNF and, not surprising, the most over-represented pathway was the apoptosis signaling. Other pathways, statistically representative of module M3 were toll-like receptor (TLR), ubiquitin proteasome, and p53 pathway. In particular, the enrichment of TLR signaling is interesting since it is well known that inducing pro-inflammatory cytokines and co-stimulatory molecules, it contributes to the development of an excessive inflammatory response, leading to both autoimmune disorders and tumor growth (67). Module M4 was mostly enriched in G-protein coupled receptor signaling, and we also observed enriched BPs related to leukocyte/lymphocyte chemotaxis, whereas the most over-represented pathway was inflammation mediated by chemokine and cytokine signaling pathways. The Wnt signaling pathway was preeminent in module M5. In addition, DEGs in this module were also primarily involved in the p53 pathway. Response to virus was the most representative BP of module M6: is worthwhile mentioning that numerous viruses have been proposed as possible triggering factors in SSc (68), and indeed, it has been estimated that up to a quarter of human tumors are connected to infection or infection-associated chronic inflammation (69).

A large number of BPs involved in immune cells activation, proliferation, and differentiation and in type I interferon signaling, were prevalent in M6. The over-represented pathways in this module were again signalings also involved in cancer and already mentioned in this analysis (i.e., p53, interleukin, PDGF, Jak/Stat, TLR, Ras, and apoptosis signaling pathway).

### MicroRNAs in SSc Sera

Systemic sclerosis is associated with an increased risk of malignancies, and in the present study, we found a modulation of genes encoding for molecules that have been previously associated to different types of cancers. An important role of miRNAs in human cancers is well established (12). The higher incidence of cancer in SSc patients prompted us to investigate whether specific cancer-related miRNAs could be deregulated in the serum of SSc patients as compared to healthy controls.

**Figure 4:**
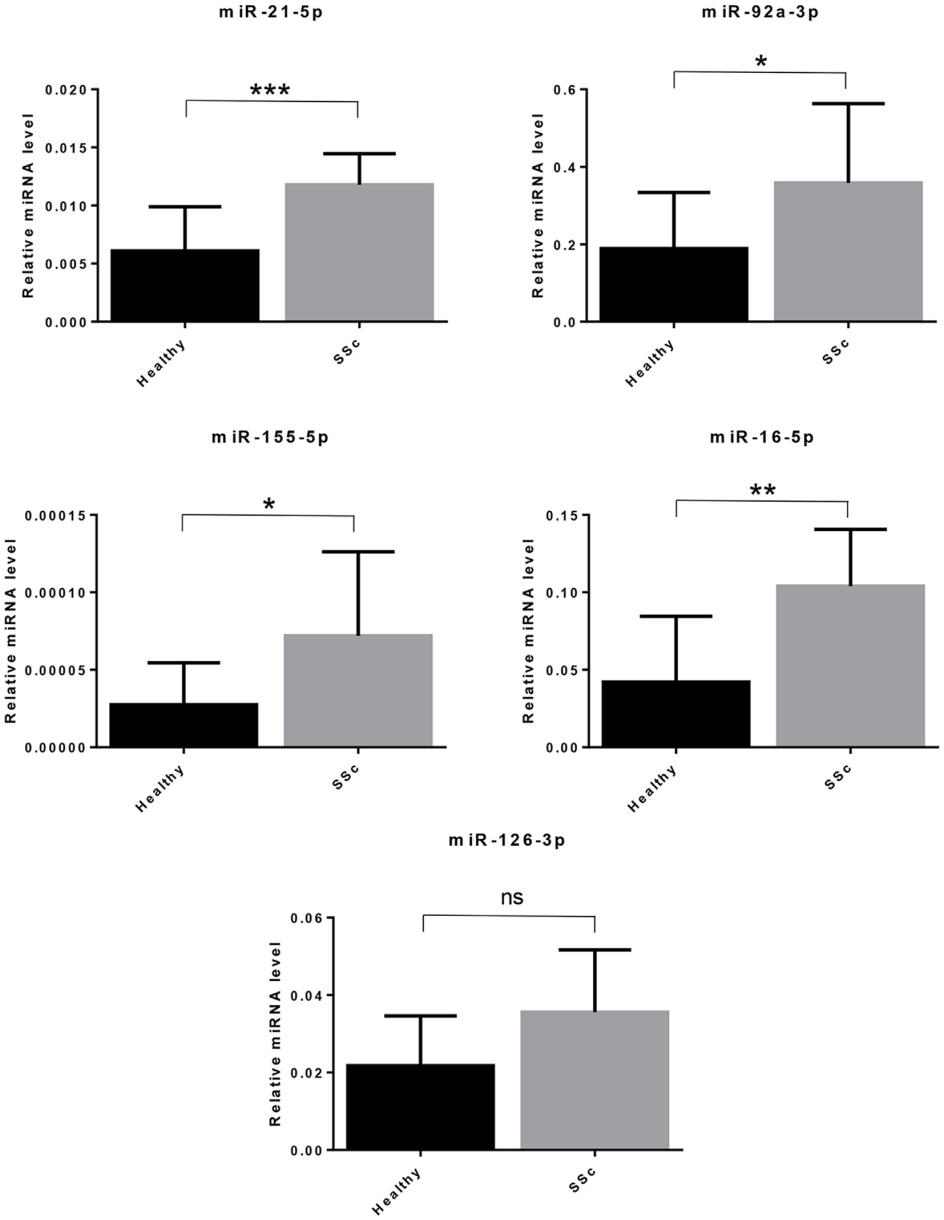
Cell-free circulating miRNA expression in systemic sclerosis (SSc) sera. The expression of the indicated miRNAs was evaluated by real-time PCR in serum of systemic sclerosis patients (*SSc*; *n* = 30) and of healthy controls (*Healthy*; *n* = 30). Expression values of mature miRNAs were calculated using the comparative ΔCt method and normalized to spike-in cel-39-3p. Histograms represent mean ± SD. *p*-values (ns = not significant; *p ≤ 0.05; **p ≤ 0.01; ***p ≤ 0.001) were determined using the Mann–Whitney rank sum test.

Since SSc patients are mainly affected by breast, lung, or hematological malignancies (70), we selected miRNAs with a solid evidence in literature for deregulation in these kind of cancers. We focused on miR-155-5p, miR-126-3p, and miR-16-5p. Furthermore, we decided to analyze miR-21-5p and miR-92a, since they play key roles in many cancers, and to confirm their upregulation in our cohort of SSc patients (Table 8). Cell-free miRNAs (cf-miRNA) in limited and diffuse SSc and in healthy sera was evaluated by real time PCR, as represented in Figure 4. A significant higher expression of 4/5 of the miRNAs tested in SSc sera was found as compared to healthy controls. miR-126-3p expression also showed a trend of upregulation in SSc samples although it did not reach statistically significant differences in the samples tested compared to controls. Moreover, no significant differences between limited and diffuse SSc were found in our analysis. miR-21-5p and miR-92a-3p were upregulated, as reported in literature, further supporting the hypothesis of their involvement in SSc pathogenesis. To our knowledge, upregulation of miR-155-5p and miR-16-5p in SSc sera has not been reported yet, and it suggests a role of these miRNAs in the disease.

**Table 8:** Cancer-related miRNAs selected for expression analysis in SSc serum.

miRNA	Modulation in cancer	Cancer	Reference
*miR-21-5p*	Upregulated	Breast; lung; leukemias	(71–73)
*miR-92a-3p*	Upregulated	Lung	(74, 75)
*miR-155-5p*	Upregulated	Breast; lung; leukemias	(71, 72, 76)
*miR-126-3p*	Downregulated	Breast	(72, 77)
*miR-16-5p*	Downregulated	Leukemias	(78)

## Discussion

Systemic sclerosis represents a major medical challenge and many unmet needs in the treatment of the disease still remain. However, advances have been made thanks to the increased understanding of the pathogenesis of SSc and the overall patients’ survival is improved.

The increased risk of malignancies in SSc is a major source of concern and the identification of risk factors for both disorders may have implications for the prognosis and for the treatment.

We therefore wanted to carry out a gene expression profile and an epigenetic analysis in scleroderma patients to verify the presence of common bases for the development of malignancies and SSc.

We have noticed that the functional classes, to which the genes modulated in limited and diffuse forms of SSc belong, were virtually overlapping and, in both cases, we observed the modulation of genes and pathways previously associated with malignancy. Although it has been reported a higher incidence of some tumors in patients with dSSc than in those with lSSc, our data seem to suggest that in both forms of the disease there is a genetic modulation that may be linked to the onset of a neoplastic transformation. Indeed, from our analysis has emerged that the various classes comprising genes potentially linked to the pathogenesis of SSc (such as apoptosis, endothelial cell activation, extracellular matrix remodeling, immune response, and inflammation), include genes that directly participate in the development of malignancies or that are involved in pathways known to be associated with carcinogenesis.

In this regard, several pathways enriched in lSSc and/or dSSc (apoptosis, glycolysis, PDGF, Fas cell surface death receptor, FAS, angiogenesis, interleukin, Ras, Jak/Stat, and EGFR signaling pathways) are involved in cancer development.

Indeed, altered apoptosis has a crucial role in the induction of a malignant phenotype, and it is well known that some oncogenic mutations block apoptosis, leading to cancer progression (79). Moreover, some cancer cells express FAS ligand (FasL) and the activation of FAS pathway may favor immune privilege to tumors by inducing apoptosis of anti-tumor lymphocytes (80).

The modulation of genes involved in the glycolytic pathway is not surprising, given that upregulation of glycolysis is a well-documented property of cancers, and this mechanism confers to tumor cells a significant growth advantage (81).

Platelet derived growth factors and PDGF receptors have substantial role in regulating cell growth during embryonal development. An enhancement of PDGF receptor signaling, may also sustain tumor cell growth. Moreover, fibroblasts, and myofibroblasts of solid tumors stroma express PDGF receptors, and PDGF stimulates these cells promoting tumorigenesis (82). Noteworthy, PDGF enhances c-myc expression and stimulates the c-myc promoter (83). This gene, found overexpressed in our lSSc dataset, coordinates cell growth and cell proliferation and its deregulation is a near-universal property of primary and metastatic cancers (84).

Ras proteins are key elements in malignant transformation; moreover, the Jak/stat signaling pathway sustains epithelial mesenchymal transition and generates a pro-tumorigenic microenvironment. The EGF receptor signaling pathway modulates migration and survival of cancer cells.

It has been established that inflammation caused by either chronic disease or infection is an important risk factor in cancer development and that a variety of interleukins are involved in both inflammation and carcinogenesis (85). In particular, among genes involved in the interleukin signaling pathways that was enriched in lSSc modulated genes, we found overexpression of the interleukin 2 receptor alpha (IL2RA) gene. The soluble form of this molecule is released from neoplastic cells and is expressed on the surface of both lymphoid and non-lymphoid cancer cells (86).

Pathway analysis also highlighted the modulation of a large number of genes (11) involved in the Type 1 interferon signaling pathway. This pathway is a hallmark of many systemic autoimmune diseases (87) including SSc (88) and has also been considered as a “double-edged sword” in cancer. Indeed, in cancer, it promotes both T cell responses and a negative feedback leading to immunosuppression. Tumor cells can take advantage from this counter-regulatory effects induced by IFN type I to avoid immune cell killing (89).

This suggests that there are multiple points of contact between the signaling pathways leading to scleroderma and those that predispose to the development of a malignancy. However, these pathways may lead to different outcomes in different tissues in which they are expressed, and indeed in patients with scleroderma, some malignancies are more frequent such as those affecting breast, lung, and lymphoid tissue.

Endothelial cell apoptosis is among the first manifestations of vasculopathy associated with the development of SSc, and fibroblasts activation and proliferation lead to the fibrotic characteristics of the disease. Gene expression profiles obtained from the PBMC of patients with SSc indicate an altered modulation of genes involved in apoptosis and in cell proliferation that could, at tissue level, exert a mitogenic effect on some cellular populations or lead to down-modulation of oncosuppressor genes, as suggested by the downregulation of CUL3 (31) in both lSSc and dSSc.

Our data also suggest the presence of a genetic modulation that can favor angiogenesis, a common feature associated to the development and progression of any type of cancer.

Modulation of many genes involved in the immune response, including genes inducible by type I interferon, reflects the strong immune system dysregulation associated with excessive antigenic stimulation typical of autoimmune diseases. Such dysregulation can either induce proliferation of cells of the immune system, leading to malignancy in susceptible individuals or modify the immune system’s regulatory mechanisms that inhibit the development of naturally occurring cancers.

Several modulated genes participate in the remodeling process of the extracellular tissue matrix. Excessive deposition of ECM promotes the development of fibrosis, hallmark of SSc, and the fibrotic and inflammatory processes of the lung have been considered the basis for the eventual onset of lung cancer in SSc. On the other hand, within these functional classes, we have also observed over-expression of metalloproteases (i.e., ADAM2) or ECM metalloproteases inducers (i.e., BSG) typically induced by several tumor cells (56, 90). Moreover, genes associated with the migration of tumor cells such as SPARC (43), a glycoprotein associated with ECM, are involved in the development of non-small cell lung cancer. Therefore, while ECM deposition and related profibrotic events may favor carcinogenesis through different mechanisms such as blocking lymphatic channels and creating niches in which carcinogens may eventually accumulate, on the other hand, there is a genetic modulation that favors cancer cells spreading.

A crucial issue in basic and clinical research is to understand gene modulation in terms of biological networks since proteins usually function in protein–protein interacting networks. We therefore wanted to dissect meaningful relationships among modulated genes in SSc, analyzing the PPI network in which their protein products can be involved. Moreover, since it is known that the deregulation of protein expression may provoke more drastic biological effects when genes/proteins with more interacting partners are involved, we focused our attention on the most connected genes/proteins that “as a matter of fact” (by definition) are included in the network areas called modules. This is particularly important when studying gene regulation in certain diseases in order to identify the molecular pathways that are most relevant in disease pathogenesis. The pathway enrichment analysis of modulated genes included in the modules confirmed the enrichment of signaling pathways (i.e., the aforementioned Jak/stat, glycolysis, Ras, PDGF, EGF receptor, Wnt, and type I interferon signaling pathways) associated with carcinogenesis in both datasets.

The genes participating to these pathways are indeed comprised in network areas (modules), whereas gene interactions are concentrated and generally underline significant biological processes.

These molecular pathways also emerged from our first global analysis of the two datasets, but the network analysis further underlined their involvement in SSc. Indeed, members of these signaling networks are concentrated in modules were the molecules that are supposed to play a prominent role in shaping the typical features of the disease, are positioned.

Another aspect we have investigated is the expression of microRNAs in SSc. Altered expression of miRNAs in SSc, as well as their involvement in inflammation and fibrosis, has been described (14, 91). Similarly, the implication of miRNAs in human cancers is well established (12). A fascinating hypothesis is that a dysregulated epigenetic control mediated by miRNAs in SSc could promote tumorigenesis. Deregulated expression of miRNAs has been found in blood of SSc patients and it could exert oncogenic effects at distant sites from SSc lesions. Indeed, the upregulation of miR-21 and miR-92a in SSc (16, 92) may support this hypothesis, since these miRNAs play a role in many tumors, repressing important oncosuppressor genes (74, 93). Thus, the higher incidence of breast, lung and hematological malignancies in SSc patients (70) prompted us to investigate whether specific cancer-related miRNAs could be deregulated in the serum of SSc patients. We found that miR-21-5p, miR-92a-3p, miR-155-5p, and miR-16-5p expression was significantly higher in SSc sera compared to healthy controls. miR-21 can play a role in SSc since it is also upregulated in SSc fybroblasts and it is implicated in TGF-β-regulated fibrosis (17). Interestingly, miR-21 upregulation promotes proliferation, migration, and invasion in lung cancer (71). Elevated miR-92a levels in serum and in dermal fibroblasts of SSc patients have also been reported (16), and this miRNA is implicated in angiogenesis and proliferation in lung cancer and it can promote leukemogenesis (74, 75). MiR-155-5p expression was also increased in SSc sera. Notably, miR-155 was found upregulated in SSc fybroblasts, and it was associated to the progression of lung fibrosis in dSSc patients (94, 95). miR-155 upregulation also sustains survival and proliferation in hematological disorders and breast cancer (72, 76). We found increased miR-16-5p levels in SSc sera despite this miRNA is frequently associated to oncosuppressor functions and it is frequently absent in human leukemias (78). Since this miRNA inhibits cell proliferation and promotes apoptosis (96), it could participate to the apoptotic process of endothelial cells, considered the first pathogenic event in SSc. A release of miR-16 in the bloodstream from apoptotic endothelial cells could explain the increased levels of the miRNA in SSc sera. We also decided to evaluate miR-126 in SSc sera since it was found downregulated in breast and lung cancer (76, 77). Moreover, miR-126 plays an important role in angiogenic signaling and in vascular integrity (97), and we found several genes involved in angiogenesis upregulated in SSc by microarray analysis. We did not find statistically significant differences in the miR-126 expression levels between SSc patients and healthy controls, although we observed a trend toward upregulation in SSc samples. In conclusion, we describe here interesting findings on deregulated cancer-related miRNAs in SSc patients. However, further studies are needed to elucidate the potential role of these miRNA in SSc. In particular, since the levels of circulating microRNAs can be affected by different cell types, interesting points will be to elucidate which cells mainly contribute to these circulating oncogenic miRNAs and whether they can play an active role in the increased tumorigenesis associated to the disease.

Taken together, our data suggest the presence of modulated genes and miRNAs that can play a predisposing role in the development of malignancies in SSc. The findings of genetic and epigenetic features that are shared by SSc and cancer shed new light on the pathogenesis of the disease and strengthen the idea that autoimmunity plays a central role in the initiation and progression of SSc, since the presence or development of malignancies is associated with particular autoantibodies. These aspects are central to a better risk stratification of patients and to develop an individualized precision medicine strategy.

## Ethics Statement

This study was carried out in accordance with the recommendations of “local ethical committee Azienda integrata Università di Verona e Ospedale Borgo Roma Verona, Italy” with written informed consent from all subjects. All subjects gave written informed consent in accordance with the Declaration of Helsinki. The protocol was approved by the “name of committee.”

## Author Contributions

APu, CL, MD, APe, and APu conceived and designed the experiments. MD, APe, and PF performed the experiments. MD, APe and APu analyzed the data. GP and ET contributed reagents. ET, GP, and CL contributed materials. MD and APe wrote the paper.

## Conflict of Interest Statement

The research was conducted in the absence of any commercial or financial relationships that could be construed as a potential conflict of interest. The reviewer SV and handling editor declared their shared affiliation.

## Footnotes

^1^http://pantherdb.org/.

^2^http://string-db.org/.

^3^www.ebi.ac.uk/arrayexpress.
